# The Temporal Extracellular Proteomics Analysis Reveals the Expression Patterns of Functional Enzymes Involved in Ramie Degumming by *Dickeya dadantii* Strain DCE-01

**DOI:** 10.3390/polym17243284

**Published:** 2025-12-11

**Authors:** Yuqin Hu, Mingqiang Gao, Xiang Zhou, Lifeng Cheng, Guoguo Xi, Si Tan, Wei Zhou, Zishu Chen, Zhenghong Peng, An Wang, Shengwen Duan, Qi Yang

**Affiliations:** 1 Institute of Bast Fiber Crops, Chinese Academy of Agriculture Sciences, Changsha 410205, China; 82101232017@caas.cn (Y.H.); gmq18785604146@163.com (M.G.); 821012430018@caas.cn (X.Z.); chenglifeng@caas.cn (L.C.); xiguoguo@caas.cn (G.X.); 15116005853@163.com (S.T.); zhouwei@caas.cn (W.Z.); chenzishu@caas.cn (Z.C.); pengzhenghong@caas.cn (Z.P.); 2 State Key Laboratory of Rice Biology and Breeding, China National Rice Research Institute, Hangzhou 310006, China; anwang010828@163.com

**Keywords:** microbial degumming of ramie, *Dickeya dadantii* strain DCE-01, proteomics, differential protein expression

## Abstract

Microbial degumming offers an environmentally sustainable route for the extraction of natural ramie fibers. However, there are currently no genetically engineered bacteria suitable for large-scale industrial production. In this study, *Dickeya dadantii* strain DCE-01, a dominant ramie-degumming bacterium, was systematically investigated using time-resolved quantitative proteomics. This research revealed a temporally coordinated, multi-enzyme catalytic mechanism, in which pectate lyases were highly expressed at the initial stage to rapidly depolymerize surface pectin, followed by sustained expression of hemicellulases that ensured the complete removal of residual non-cellulosic materials. Quantitative real-time PCR (qRT–PCR) results showed strong concordance with the proteomic data, confirming that transcriptional regulation drives the dynamic enzymatic response. This integrative analysis, linking macroscopic morphological evolution with microscopic molecular changes, elucidates the intrinsic mechanism underlying efficient biological degumming by *D. dadantii* strain DCE-01 and provides valuable insights for the rational design of high-performance engineered bacteria for industrial fiber processing.

## 1. Introduction

Ramie (*Boehmeria nivea*), a perennial bast fiber crop, is among the most important natural sources of cellulose. Its fibers are renowned for their exceptional strength, flexibility, moisture absorption, dyeability, and inherent antibacterial properties, making them highly valuable for textiles, composites, and biotechnological applications [1,2]. Structurally, ramie fibers contain about 70% cellulose and approximately 30% non-cellulosic “gum,” consisting mainly of pectin, hemicelluloses (xylans and mannans), and minor amounts of lignin [3]. These gummy substances adhere to the fiber bundles, hindering separation and extraction, thus making degumming a critical step in producing high-quality fibers. Traditional alkaline chemical degumming remains the industrial standard but often causes fiber damage, high energy consumption, and environmental pollution [4]. In contrast, microbial degumming has emerged as a sustainable alternative that relies on microbial enzymes to hydrolyze non-cellulosic materials. This method achieves effective gum removal while preserving cellulose integrity and offers operational simplicity, reduced environmental impact, and lower cost [5,6,7,8,9].

*Dickeya dadantii*, a Gram-negative bacterium and a member of the genus *Dickeya*, secretes a suite of extracellular enzymes, such as pectinases, hemicellulases, and proteases. These enzymes collectively degrade the polysaccharide components of plant cell walls. This characteristic makes it an excellent choice for microbial degumming processes. Researchers isolated strain DCE-01 from *D. dadantii*, which efficiently removes gum without affecting the original fiber structure. They obtained recombinant enzymes by heterologous expression of their unique xylanase gene in *E. coli* for application in ramie degumming [10]. However, the activity of extracellular enzymes secreted by strain DCE-01 during degumming does not always match the gum composition of ramie fibers. This leads to unstable degumming effects and high residual gum content, issues that require further resolution. Therefore, analyzing and identifying the dynamic expression patterns of different extracellular enzymes produced by strain DCE-01 during degumming and precisely matching them to different ramie gum components is crucial for optimizing the microbial degumming process [11].

Proteomics provides a powerful approach to elucidate microbial enzyme expression under different environmental conditions [12,13]. Previous temporal proteomic studies have revealed dynamic protein regulation during biofilm formation in *Burkholderia thailandensis* [14] and metabolic adaptations in *Bacillus subtilis* during ramie bast degumming [15]. Nonetheless, the global and time-resolved proteomic responses of *D. dadantii* strain DCE-01 during degumming remain largely unexplored.

In this study, a time-resolved biodegumming model of ramie fibers using *D. dadantii* strain DCE-01 was established and combined with quantitative proteomic and transcriptional analyses. This integrated approach aimed to elucidate the dynamic expression patterns of extracellular enzymes, reveal synergistic interactions among key functional enzymes, and correlate proteomic changes with fiber morphological evolution. The findings provide new insights into the intrinsic mechanisms of microbial degumming and a theoretical foundation for developing high-efficiency engineered bacteria for industrial ramie processing.

## 2. Materials and Methods

### 2.1. Experimental Materials

The ramie was purchased from Yuanjiang City, Hunan Province, China. The bast of the ramie used for the experimental analysis was the 1st variety of ramie (bast fiber crop) cultivated by the Yuanjiang Comprehensive Experimental Station of the Chinese Agricultural Research System. The ramie bark was manually peeled off the core and dried without mold.

The *D. dadantii* strain DCE-01 was selected from the strain library of our laboratory and was used as the degumming strain of ramie bast. The chemical components of the bast of ramie include cellulose (78.55%), hemicellulose (12.51%), pectin (4.04%), lignin (2.43%), and water-soluble substances (2.47%) [16].

### 2.2. Incubation Media

Liquid activation medium (LB): 1.0% glucose, 0.5% peptone, 0.5% meat extract, 0.5% NaCl.

Nutrient agar medium: 10% peptone, 0.5% NaCl, 0.3% beef extract.

### 2.3. Strain Activation

The *D. dadantii* strain DCE-01 was activated in 5 mL of liquid medium at 34 °C, 180 r/min for 18 h of culture. Then, a single colony was picked with a disposable inoculation loop and streaked onto a plate. The plate was placed in a 34 °C incubator for 18 h of culture. After colonies grew on the plate, a single colony was picked with an inoculation loop and transferred to 5 mL LB medium at 34 °C, 180 r/min for 8 h to obtain the degumming seed liquid. Finally, the seed liquid was transferred to a 2% to 100 mL conical flask for expansion for 6 h to obtain the final DCE degumming liquid, which can be used in the ramie degumming system.

### 2.4. Ramie Fiber Degumming Treatment

A total of four degumming time points of 0 h, 8 h, 16 h, and 24 h were selected. 100 mL of water and 10 g of ramie were placed in a test tube, and the sterilization conditions were 121 °C, 20 min. The degumming liquid obtained after 6 h of expansion was added to the ramie system at a 2% ratio, and the culture conditions were 34 °C, 180 r/min. Each sample was set with 3 replicates. The fermentation liquid at 0 h, 8 h, 16 h, and 24 h was extracted, and the pH value of the fermentation liquid was measured using an acid-base detection instrument. Then, the fermentation liquid was centrifuged at 3000 r/min for 10 min, the precipitate was discarded, and the supernatant was retained for protein extraction.

### 2.5. Post-Treatment of Ramie Fiber

All ramie fiber samples were washed with water to remove the gum that had degraded but still adhered to the fibers during degumming, and then dried at 60 °C to a constant weight to determine the weight loss rate or residual gum content.

The weight loss rate R of ramie fiber was calculated as follows [17]: The ramie fiber samples before and after degumming were dried at 60 °C to a constant weight, with the weight values being M0 and M1. The weight loss rate R was derived from Equation (1):

R = (M0 − M1)/M0 × 100%(1)

The residual gum content C of the ramie fiber was determined as described previously [18], that is, after degumming of the ramie fiber samples at 60 °C, the weight value was W0; then, the ramie fiber was added to a fixed condenser-Allihn type, 200 mL of water and 20 g/L NaOH were added, and the system was boiled for 2 h. The residue was washed and dried at 60 °C to a constant weight, with the weight value being W1. The residual gum content was calculated using Equation (2):

C = (W0 − W1)/W0 × 100%(2)

The single fiber breaking strength value of the treated ramie fibers was evaluated in reference [19,20].

### 2.6. Cryo-Scanning Electron Microscopy Analysis

A small amount of ramie fiber sample was taken from each time point (0 h, 8 h, 16 h, 24 h) after degumming and drying to constant weight, and placed on the conductive glue. Then, the sample was sprayed with gold using an ion sputtering coating machine for 70 s. Afterwards, the surface features of the sample were observed under a scanning electron microscope (Hitachi SU-3500, Hitachi, Tokyo, Japan) at a voltage of 15 kV, and photos were taken and recorded.

### 2.7. Protein Extraction and Quality Detection

The specific steps for protein extraction in this experiment are as follows: First, the sample was dissolved in a water bath at room temperature. After complete dissolution, 10 mL was taken and placed in a 3 KDa ultrafiltration centrifuge tube. The centrifuge tube was placed at 4 °C, and a 6400× *g* centrifuge was used for 40 min. Repeat this step until all the samples are collected in the ultrafiltration tube. The ultrafiltration tube was washed with 200 μL of chromatography water at least 3 times to mix with the filtrate. The above mixture was centrifuged at 4 °C, 12,000× *g* for 15 min, and the supernatant was reserved. The overnight freeze-dried supernatant was dissolved in SDS lysis buffer at room temperature for 3 min, then the solution was centrifuged at 4 °C, 12,000× *g* for 10 min, and the supernatant was collected. After protein concentration determination, the supernatant was stored at −80 °C.

When conducting the SDS-PAGE experiment, to better separate the proteins, the concentrations of the separating gel and the concentrating gel were selected as 12% and 5%, respectively. Electrophoresis was conducted at room temperature for 75 min. After the electrophoresis, use the eStain LG protein staining instrument (GenScript, Piscataway, NJ, USA) for Coomassie brilliant blue staining. Use the gel image analysis system to perform imaging analysis on the stained gel, and adjust the system imaging coefficient to make the protein bands clearly observable.

### 2.8. Trypsin Digestion

100 μg of the extracellular protein samples were taken, and each group of samples was diluted to the same concentration and volume using SDS lysis buffer. DL-Dithiothreitol (DTT) was added to the diluted protein solution, and the final concentration of DTT was adjusted to 5 mM. Mix well and incubate at 55 °C for 30 min, then immediately cool to room temperature on ice. Then, add iodoacetamide to the cooled protein solution until the concentration reaches 10 mM. Mix well, place at room temperature in the dark for 15 min, then add 6 times the volume of acetone to precipitate the protein. Store overnight at −20 °C. After removing the protein samples, centrifuge at 4 °C, 8000× *g* for 10 min, collect the precipitate, let it stand for 3 min, then add 100 μL of Triethylammonium bicarbonate buffer (TEAB solution, concentration of 200 mM) and 1 mg/mL of Trypsin-TPCK. Digest overnight at 37 °C, freeze-dry the digestion sample, and store at −80 °C.

### 2.9. Tandem Mass Tags (TMT) Labeling

Take out the stored protein samples and add 100 μL of 100 mM TEAB buffer to them. Mix them slowly by pipetting. Take 40 μL and add it to a 1.5 mL Ep tube. Take the TMTpro reagent from the refrigerator and place it on ice to thaw to room temperature. Then, accurately add 20 μL of anhydrous acetonitrile to the thawed reagent and mix and centrifuge. Use a pipette to accurately measure 10 μL of the centrifuged solution and add it to the protein sample. Shake several times and let it stand at room temperature for 1 h. Then, add 5 μL of 5% hydroxylamine to terminate the reaction. Store the frozen protein samples in a −80 °C ultra-low temperature refrigerator. The detection results of the labeling efficiency show that the labeling efficiency is 99.48%, which is greater than 97%, and meets the quality control standards.

### 2.10. Chromatography and Mass Spectrometry Conditions

(1) Chromatography Conditions

Load the samples onto the pre-column calibrated PepMap 100 μm × 2 cm, with a flow rate of 300 nL/min. Then, separate through the analytical column calibrated PepMap Rsplc 75 μm × 15 cm. Phase A: H_2_O-FA (99.9: 0.1, *v*/*v*/*v*); Phase B: ACN-H_2_O-FA (80:19.9:0.1, *v*/*v*/*v*); Gradient elution conditions: 0–40 min, 5–30% B; 40–54 min, 30–50% B; 54–55 min, 50–100% B; 55–60 min, 100% B.

(2) Mass Spectrometry Conditions

The first-level mass spectrometry mass resolution was set at 70,000 (ppm), the automatic gain control value was set at 1 × 10^6^, the maximum injection time was 50 MS, and the mass spectrometry scan was set to full scan mass-to-charge ratio within the range of 300–1600 *m*/*z*. Collision energy was set at 32; MS/MS resolution was set at 35,000, the automatic gain control was set at 2 × 10^5^, the maximum ion injection time was 80 ms, and the dynamic exclusion time was set at 15 s.

### 2.11. Data Analysis and Database Search

Data is analyzed using Xcalibur 2.1.2 software (Thermo-Fisher Scientific Company, Waltham, MA, USA), using Proteome Discover 2.4 as the search software, using the Sequest HT algorithm, and searching for spectra in the Uniprot database. Based on the relative abundance of the reported ions of different TMT reagents, the relative quantitative results of proteins are calculated. In the two comparable groups, the ratio of protein values changes by more than 1.2 or less than 0.83, and the *p* value is less than 0.05, indicating a significant difference, while other peptide ions were used for protein identification. Database search conditions: enzyme cleavage type selected as Trypsin, Instrument set as Q Exactive, MS1 tolerance set at 10 ppm, MS2 tolerance set at 0.02 Da, the maximum number of missed cleavage sites allowed (Max.Missed Cleavage Sites) set to 2. Variable modification type: oxidative/+15.995 Da, dynamic TMT 10 plex/+229.163 Da; N-terminal modification type as acetyl group/+42.011 Da and TMT10plex/+229.163 Da, the specific fixed modification type as carbamoylmethyl group/+57.021 Da.

### 2.12. Bioinformatics Analysis

GO enrichment analysis (Gene Ontology/www.geneontology.org, accessed on 1 March 2025) was an important method for protein function annotation. It mainly annotated the existing protein data from three aspects: biological process, cellular composition, and molecular function. The differentially expressed proteins were tested using Fisher’s exact two-tailed test, and the significance was determined based on the criterion that the *p*-value is less than 0.05. KEGG was a major public database used for systematic analysis of protein pathways (https://www.kegg.jp/, accessed on 1 March 2025). Through pathway analysis, the main metabolic pathways and signal transduction pathways in which proteins are involved can be determined. The differentially expressed proteins were screened based on the criterion of a fold change of 1.5 times and a *p*-value less than 0.05.

### 2.13. Quantitative Real-Time PCR

Quantitative real-time PCR (qRT-PCR) amplification, detection, and analysis were performed with a StepOne Plus Real-Time PCR System (ABI, Foster, CA, USA) using 2× SG Fast qPCR Master Mix (High Rox, B639273, BBI). The transcriptional levels of six proteins were selected and analyzed by qRT-PCR (P18209, P0C1A9, Q9X6Z2, P01CA7, P11073, and P04959). The six genes for qRT-PCR validation were selected from the pool of differentially expressed proteins (DEPs) based on a combination of biological relevance and statistical significance. First, we focused on DEPs functionally annotated as pectinases, given the pivotal role of pectin degradation in initiating the ramie degumming process. To ensure an objective and unbiased representation of the most substantial changes, we then shortlisted the top six pectinase-encoding genes that exhibited the most significant alterations, using the stringent thresholds of a *p*-value < 0.05 and an absolute fold change (FC) > 2. This two-tiered strategy ensured that the validated genes were both functionally crucial to the degumming mechanism and among the most prominently regulated at the proteomic level. The transcript generated from the 16S rRNA gene was used as the endogenous housekeeping gene control for normalizing the expression levels of the target transcripts, and relative gene expression levels were calculated using the widely established comparative Ct (2^(−ΔΔCt)^) method [21]. All sequences of the primers used in real-time PCR were developed with Primer version 5.00 and listed in Table S2.

## 3. Results

### 3.1. SEM Scanning Electron Microscopy Analysis

**Figure 1:**
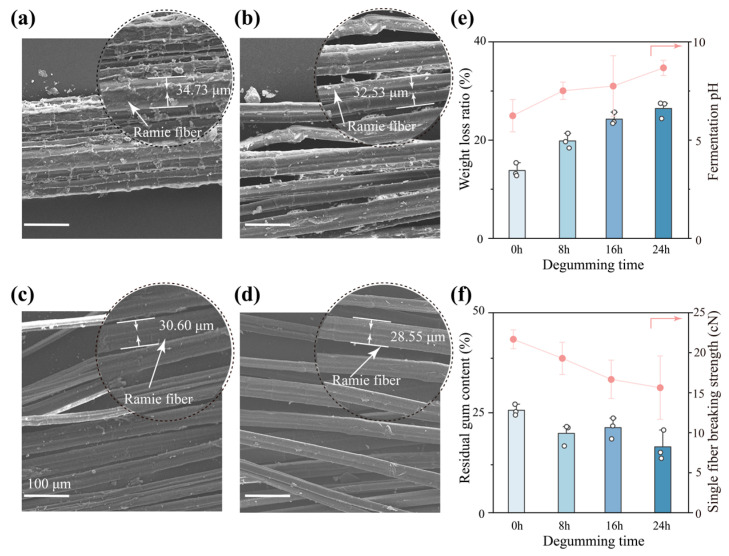
SEM analysis of fibers at different degumming time points, weight reduction rate, fermentation broth pH, residual fiber rate, and single-fiber strength. (**a**) SEM image of fibers at 0 h of degumming; (**b**) SEM image of fibers at 8 h of degumming; (**c**) SEM image of fibers at 16 h of degumming; (**d**) SEM image of fibers at 24 h of degumming; (**e**) Weight reduction rate and fermentation broth pH of ramie fibers at different degumming time points, *n* = 3; (**f**) Residual gum content and single-fiber strength of ramie fibers at different degumming time points, *n* = 3.

The ramie fibers were observed under a scanning electron microscope (SEM) at four different degumming periods (0 h, 8 h, 16 h, and 24 h) by the *D. dadantii* strain DCE-01. The appearance and fiber morphology of ramie at different degumming periods were analyzed. As shown in Figure 1a, the surface of the un-degummed ramie bast fibers presented obvious roughness and a large amount of non-cellulose substances covering the fibers. The fiber bundles were closely adhered to each other and were difficult to separate. After 8 h of degumming by *D. dadantii* strain DCE-01 (Figure 1b), the adhered substances on the fiber surface began to decrease, and the fiber bundles showed initial signs of loosening. With the extension of degumming time to 16 h (Figure 1c), the gum substances on the fiber surface were significantly removed, and the fiber bundles further dispersed into finer single fibers, with a significant improvement in surface smoothness. When the degumming time reached 24 h (Figure 1d), the fiber bundles had completely separated into independent single fibers, with a clean and smooth surface, and almost no gum substances remained. In addition to the qualitative morphological observations, the average diameter of the ramie fibers was quantified from SEM images (*n* = 5). As summarized in Table S1, the fiber diameter decreased progressively from approximately 32 μm (0 h) to 24 μm (24 h) with increasing degumming duration. This reduction reflects the gradual removal of the outer gum layer and enhanced exposure of cellulose fibrils, indicating that the *D. dadantii* strain DCE-01 exhibits an efficient degumming capability that facilitates subsequent fiber extraction and processing.

We quantified the removal of gum substances during the degumming process and analyzed the changes in the acid-base environment of the microbial fermentation environment. As shown in Figure 1e, the weight loss rate of ramie fibers increased steadily with the extension of degumming time. From approximately 15% at 0 h to approximately 30% at 24 h, this indicates that the *D. dadantii* strain DCE-01 continuously and effectively degraded the non-cellulose substances in the ramie fibers throughout the degumming process. At the same time, the pH value of the degumming solution showed an upward trend, from approximately 6.5 at 0 h to approximately 8 at 24 h. This pH increase may be attributed to the degradation of pectin and hemicellulose by the *D. dadantii* strain DCE-01. Pectin is a complex acidic polysaccharide with a large number of galacturonic acids on its molecular chain, which makes the initial degumming system acidic [22]. As the acidic substances (pectin) are gradually hydrolyzed, the hydrolytic components in the fermentation solution decrease, resulting in a gradual increase in pH value.

In addition, we further analyzed the degumming effect of the *D. dadantii* strain DCE-01 and evaluated the impact of the degumming process on the mechanical properties of the fibers. As shown in Figure 1f, the residual gum rate of ramie fibers decreased significantly with the extension of degumming time, from approximately 26% at 0 h to approximately 18% at 24 h. This is highly consistent with the microscopic morphology observation results (Figure 1a–d) and the weight loss rate data (Figure 1e), further confirming the excellent degumming efficiency of the *D. dadantii* strain DCE-01. However, it is worth noting that as the gum substances were removed, the strength of individual fibers showed a downward trend from approximately 22.5 cN at 0 h to approximately 16 cN at 24 h. This may be because these gum substances played a role in connecting microfibers, making the ramie fibers more closely bound. When these gum substances were removed, the binding between fibers became less tight, and this structural change may lead to a reduction in mechanical connections between microfibers, resulting in a decrease in fracture strength [23]. Therefore, in practical applications, the degumming time needs to be optimized to ensure adequate degumming while maximizing the retention of the mechanical properties of the fibers.

The above research content indicates that the *D. dadantii* strain DCE-01 exhibits significant degumming effects during the degumming process of ramie bast, effectively removing the gum substances on the fiber surface, allowing the fiber bundles to be fully separated, and reducing the residual gum rate to approximately 10% within 24 h. This process is accompanied by a decrease in the pH value of the degumming solution, suggesting that the *D. dadantii* strain DCE-01 may achieve effective degradation of the gum substances in ramie through acid production and/or the secretion of related hydrolases (pectinase, hemicellulase), working in synergy.

### 3.2. Dynamic Changes in the Protein Profile of D. dadantii Strain DCE-01

**Figure 2:**
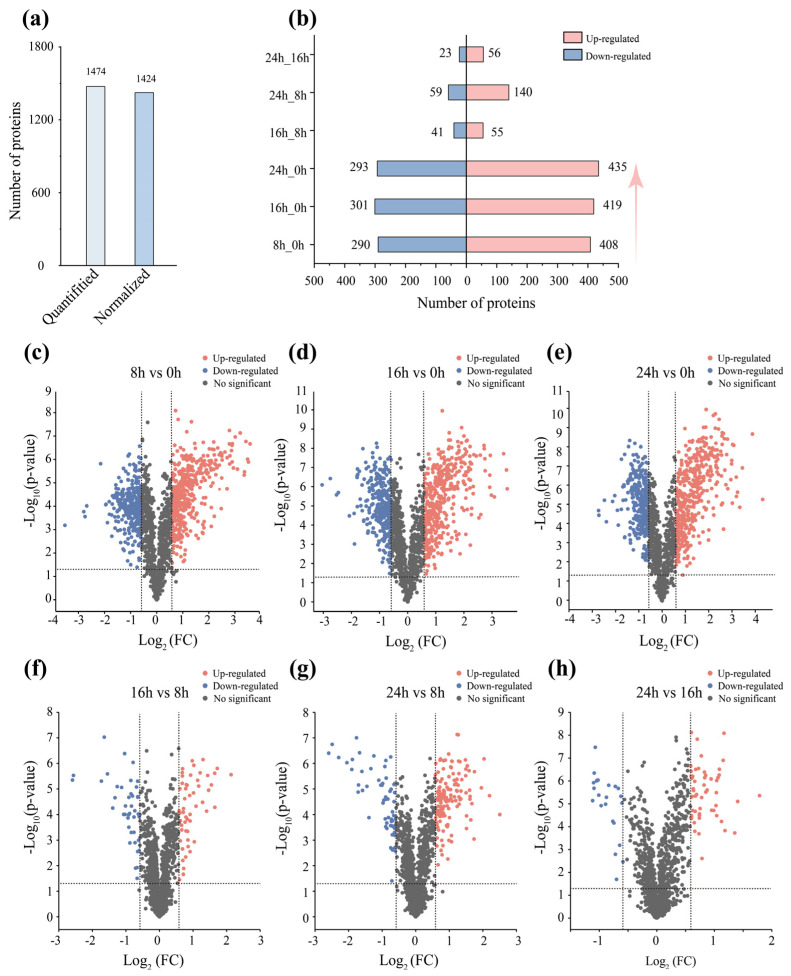
Dynamic changes in the protein profile of *D. dadantii* strain DCE-01. (**a**) Quantitative number of proteins and the number of differentially expressed proteins after normalization. (**b**) Upregulated and downregulated differentially expressed protein numbers at different degumming stages. (**c**–**h**) Volcano plots of differentially expressed protein numbers at different time points.

To systematically evaluate the physiological response of *D. dadantii* strain DCE-01 during the degumming process, we conducted quantitative analysis of the protein expression profiles at different treatment time points (8 h, 16 h, 24 h) using TMT proteomics technology. A total of 1474 proteins were identified (1a). In this study, we compared different time points with the initial state (0 h) to reveal the protein expression dynamics of the bacteria during degumming, and it was found that the number of differentially expressed proteins (DEPs) at 8 h, 16 h, and 24 h remained at a relatively high efficient level (698, 720, and 728, respectively), as shown in Figure 2b. This indicates that the protein profile of *D. dadantii* strain DCE-01 is highly active throughout the degumming process to adapt and utilize the environment of linen bast degumming.

To further analyze this dynamic change, we plotted volcano plots of protein expression differences between different treatment time points, visually demonstrating the significance and fold change of protein expression differences. As shown in Figure 2c, a large number of proteins showed significant upregulation or downregulation in the comparison between 8 h vs. 0 h, indicating that the *D. dadantii* strain DCE-01 rapidly adjusted its protein profile to adapt to the substrate environment and initiate the degumming process in the early stage of degumming. As the degumming time extended to 24 h (Figure 2e, 24 h vs. 0 h), the number and magnitude of differentially expressed proteins reached the maximum, especially the upregulated proteins, whose Log2 (FC) and −Log10 (*p*-value) values significantly increased, which is consistent with the continuous improvement of the degumming effect. However, in the comparisons during the degumming process (Figure 2f, 16 h vs. 8 h; Figure 2h, 24 h vs. 16 h), the number of significantly differentially expressed proteins decreased significantly, and the vast majority of proteins were concentrated in the central area of the volcano plot, indicating that the protein expression profile of *D. dadantii* strain DCE-01 tended to stabilize in the later stages of degumming, and the core degumming mechanism may have been operating efficiently.

Therefore, the proteomic results of this study clearly reveal the staged dynamic response mechanism of *D. dadantii* strain DCE-01 during the linen degumming process. The strain rapidly adapts to the environment and initiates degumming through a large-scale protein profile in the initial stage (0 h–8 h). Subsequently, in the 8 h–16 h period, the protein expression profile tends to stabilize and enter an efficient and sustainable degumming mode. These findings provide important proteomic references for understanding the intrinsic mechanism of bacterial biodecorating and optimizing industrial degumming processes.

### 3.3. Differential Protein Expression Analysis

The GO and KEGG enrichment analyses were conducted on the differential proteins of the *D. dadantii* strain DCE-01 at different time points (0 h, 8 h, 16 h, and 24 h) during the ramie degumming process, revealing a clear and staged physiological and metabolic pattern of this strain during the degumming process. The entire process can be divided into three key stages: preparation, mid-stage, and late-stage. From 0 h to 8 h, although the degumming reaction had just begun, the *D. dadantii* strain DCE-01 had already shown significant physiological responses. The GO analysis indicated that the differential proteins were mainly enriched in the “molecular function” category, particularly “FMN binding” and “metal ion”, suggesting that the bacteria were actively synthesizing or activating their enzyme systems, as FMN and metal ions are key cofactors for many redox enzymes and hydrolases, which are crucial for subsequent complex enzyme-promoting reactions and degumming [24,25]. Meanwhile, the KEGG enrichment analysis revealed that “ribosome” was highly enriched at 8 h. The ribosome is the core machine for protein synthesis, and its enrichment directly reflects the large-scale protein production of the cell, preparing for adapting to the new environment and initiating degumming [26]. Additionally, the appearance of “pentose and glucuronic acid interconversion” indicates that the bacteria have begun to utilize relatively simple sugars in the relatively simple ramie as their initial energy source.

After 16 h of degumming, the metabolic activity intensity of *D. dadantii* strain DCE-01 significantly increased. The GO analysis results showed that the enriched functions shifted from the molecular level to the complex “biological process”. Multiple kinase and nucleotide biosynthesis processes were significantly enriched, such as “de novo purine nucleotide biosynthesis process” and “leucine biosynthesis”. The activation of these anabolic networks reflects increased energy and precursor demands associated with cell growth, ribosome assembly, and enzyme production, which indicates that *D*. *dadantii* strain DCE-01 underwent an anabolic transition toward accelerated growth and macromolecular synthesis, establishing the metabolic and enzymatic foundation required for efficient ramie fiber degumming [27]. The results of the KEGG analysis were also consistent with this. In addition to the efficient and continuous high-level enrichment of “ribosome”, “arginine biosynthesis” and “aldol and dicarboxylic acid metabolism” also increased, indicating that the bacteria were using various carbon sources to synthesize the components they needed to support their high-intensity growth and enzyme production [28]. At 24 h of degumming, the physiological activities of *D. dadantii* strain DCE-01 entered a coordinated peak. One key finding of the GO analysis was the enrichment of “quorum sensing”. This indicates that as the density of the bacterial community increased, individual bacteria began to communicate information and might synchronously activate specific gene expression [29]. This strategy optimizes the degumming enzyme strategy, ensuring the degumming period is carried out at the optimal time with the highest efficiency. The results of the KEGG analysis also confirmed this. The continuous and significant enrichment of “ribosome” again emphasized that high-level protein synthesis was the main feature of this stage. These newly synthesized proteins might be mainly used for efficient degumming enzyme systems.

**Figure 3:**
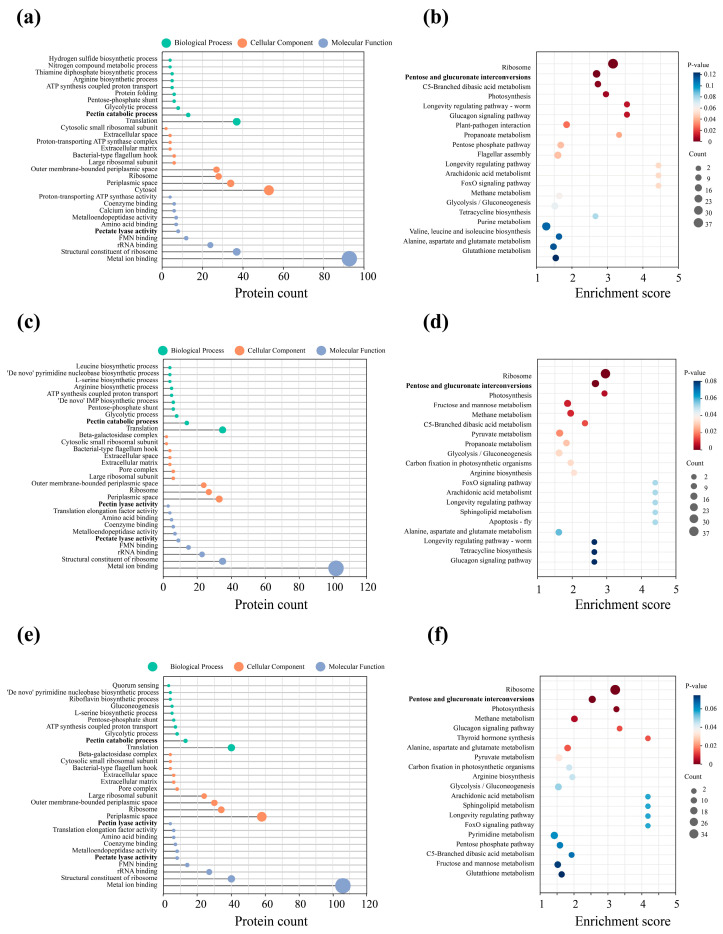
KEGG analysis and GO analysis of the differential proteins in the different degumming time comparison groups: 8 h_0 h, 16 h_0 h, and 24 h_0 h. (**a**,**b**) are the KEGG analysis and GO analysis of the differential proteins in the 8 h_0 h comparison group; (**c**,**d**) are the KEGG analysis and GO analysis of the differential proteins in the 16 h_0 h comparison group; (**e**,**f**) are the KEGG analysis and GO analysis of the differential proteins in the 24 h_0 h comparison group. The pathways highlighted in bold are those closely associated with the degumming process, including “pectin catabolic process”, “pectate lyase activity”, and “pentose and glucuronate interconversions”. They represent the core biological changes across the different degumming time points.

In conclusion, the mechanism of action of the *D. dadantii* strain DCE-01 in the ramie degumming process is not a single enzyme action process, but a dynamic biological process with the coordinated action of multiple enzymes. These findings provide an important theoretical basis for understanding the intrinsic mechanism of bacterial biodecoration and optimizing industrial application processes (Figure 3).

### 3.4. Differential Protease Spectrum Analysis

**Figure 4:**
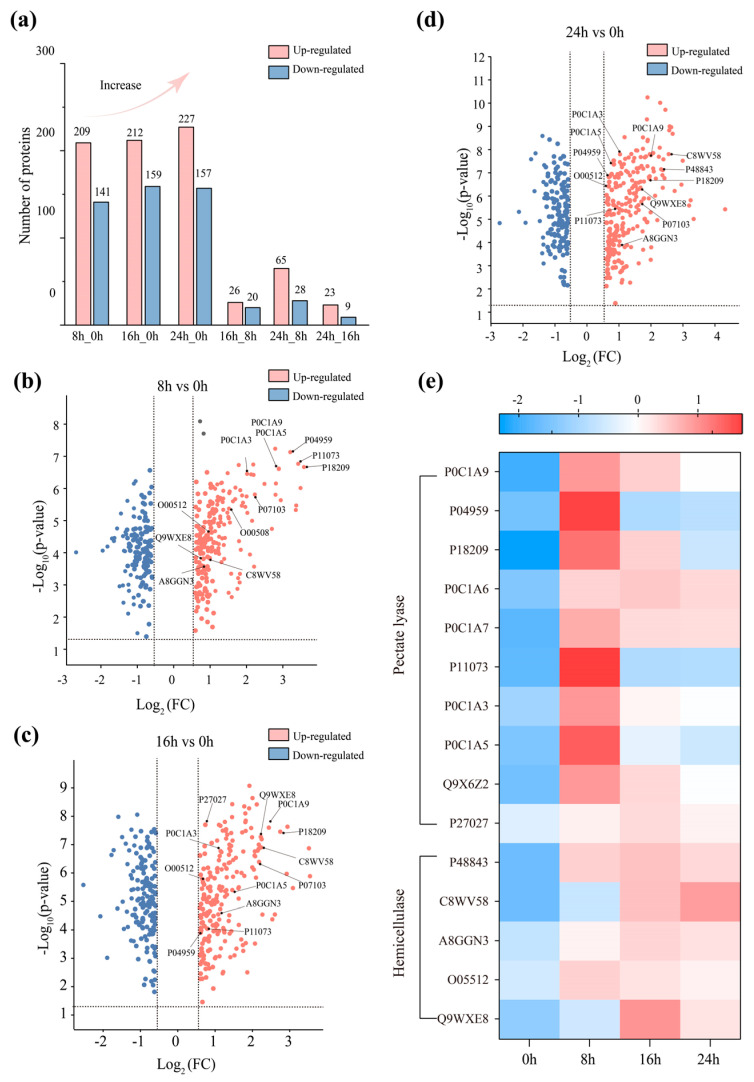
Analysis of proteases at different degumming time points and related degumming enzyme analysis. (**a**) Analysis of the number of upregulated and downregulated proteases at different degumming periods; (**b**–**d**) Volcano plots of proteases at different degumming periods; (**e**) Heatmap analysis of related degumming enzymes.

Among the 1424 proteins that have been quantified, up to 792 were identified as enzymes, indicating that the *D. dadantii* strain DCE-01 secreted a large amount of enzyme substances during the degumming process. Figure 4a shows the statistical count of differentially expressed protein enzymes at different degumming time points. Compared with 0 h degumming, significant protein enzyme differential expression was detected in the fermentation solutions at 8 h, 16 h, and 24 h, and the number of expressed proteins significantly increased, suggesting that the *D. dadantii* strain DCE-01 continuously adjusted its protein expression profile to adapt to the degumming environment and promote the degradation of the gum. However, the comparison in the subsequent degumming process showed that the number of differentially expressed protein quantities decreased significantly in 16 h vs. 8 h (26/20) and 24 h vs. 16 h (65/28), indicating that the protein expression of the *D. dadantii* strain DCE-01 tended to stabilize in the later stage of degumming.

The protein enzymes expressed at different times were analyzed by a volcano plot for up-regulation and down-regulation, and 15 degumming enzymes related to the ramie degumming process were selected (Figure 4b–e). They mainly consist of two categories: pectin lyase and hemicellulase, including 1 pectin methylesterase, 9 pectinases, 1 mannanase, 1 xylanase, and 3 galactosidases. The expression levels of 15 degumming enzymes at different times were visualized by a heat map, clearly showing their dynamic changes (Figure 4e). At 8 h of degumming, the expression levels of most pectin lyases (Pectate lyase) were significantly upregulated. This indicates that the strain quickly concentrates resources on synthesizing these enzymes when it first comes into contact with the bast of the ramie, to efficiently break down the pectin between the fiber bundles. For example, the expression ratio of pectinase P18209 (PelD) was as high as 11.956 times, making it the core component in the initial degumming. At 16 h, although the expression levels of some pectinases remained at a high level, their expression ratios were generally lower than those at 8 h. This indicates that after most of the pectin was removed, the synthesis strategy of the strain began to adjust. Meanwhile, hemicellulase (Hemicellulase) began to play a more important role at this stage. Although their overall up-regulation may not be as high as pectinases, their continuous high expression ensured the clearance of residual hemicellulose. The expression level of C8WV58 (β-galactosidase) continued to rise at 16 h and 24 h. At 24 h, the expression levels of most pectinases significantly decreased, and the color of the heat map became lighter or even blue, indicating that the strain had basically completed the main degumming task, but the expression level of hemicellulase (such as C8WV58) continued to maintain or reach the peak at this stage, indicating that they were still expressed in the final stage of degumming. Pectin lyase is the main enzyme for degrading pectin, and pectin is one of the main non-cellulose gums in the bast fibers of ramie [30]. They cleave the α-1,4-D-galacturonic acid glycosidic bonds in the main chain of pectin molecules through β-elimination reactions, thereby breaking down long-chain pectin into small molecules, allowing the fiber bundles to be separated [31]. Pectin methyl esterase (pemA, P0C1A9) had a high expression level at the beginning of degumming (7.410 times), and its function may be to help pectin lyases better approach and decompose pectin. Pectin lyase proteins, such as PelD (P18209) and PelC (P11073), showed the highest expression difference ratios at the early stage of degumming (8 h vs. 0 h), which were 11.956 and 10.710, respectively. This indicates that when the *D. dadantii* strain DCE-01 comes into contact with ramie fibers, it rapidly synthesizes a large amount of pectinase to quickly break down the pectin between the fiber bundles. Interestingly, as the degumming time increases, the expression levels of many pectinolytic enzymes show a downward trend. In the comparisons of 16 h vs. 8 h and 24 h vs. 16 h, the fold differences in the expression of many enzymes are far less than 1.0, which indicates that in the middle and later stages of degumming, the strain has completed most of the pectin degradation tasks and reduced the synthesis of related enzymes. This demonstrates the efficient strategy of the strain in resource utilization.

Hemicellulase is mainly responsible for degrading hemicellulose, which is another major gum in the bast fibers of ramie. These enzymes usually work in synergy with pectinase to complete the degumming process [32]. Among them, galactosidase (P48843, C8WV58, A8GGN3) shows a continuously increasing expression level throughout the degumming process, indicating that the degradation of hemicellulose may be a continuous step throughout the entire degumming process. Among them, C8WV58 and P48843 start to increase their expression levels at 8h and 16h, but the degree of increase may be slightly lower than that of pectinase. This may suggest that the decomposition of hemicellulose may be a secondary or accompanying process, and the strain is secreting hemicellulase to cooperate in the decomposition of hemicellulose while decomposing pectin. Interestingly, the expression level of C8WV58 reaches the highest (5.870 times) at 24 h, indicating that even in the later stage of degumming, the strain is still continuously removing residual hemicellulose to achieve the best degumming effect. Mucopolysaccharidase O05512, although its expression level is relatively low, its presence indicates that the *D. dadantii* strain DCE-01 has a comprehensive enzyme system capable of decomposing various types of hemicellulose, thus ensuring the thoroughness of degumming. Xylan is a common hemicellulose in plant cell walls, and xylanase Q9WXE8 has the highest expression level at 16h (4.746 times), indicating that the strain hydrolyzes xylan at the middle stage of degumming to enhance the decomposition of xylan.

In conclusion, the degumming ability of the *D. dadantii* strain DCE-01 is not dependent on the action of a single enzyme, but is a complex process of the cooperation of multiple pectinases and hemicellulases. Bacteria rapidly and abundantly synthesize pectinases in the early stage of degumming, responsible for quickly opening the fiber bundles; then they synthesize hemicellulases to continuously decompose the residual hemicellulose, ensuring the thorough removal of the gum. This mechanism of multi-enzyme division of labor and dynamic regulation is precisely the key to the strain achieving efficient biological degumming.

### 3.5. Quantitative RT-PCR of Key Transcripts

**Figure 5:**
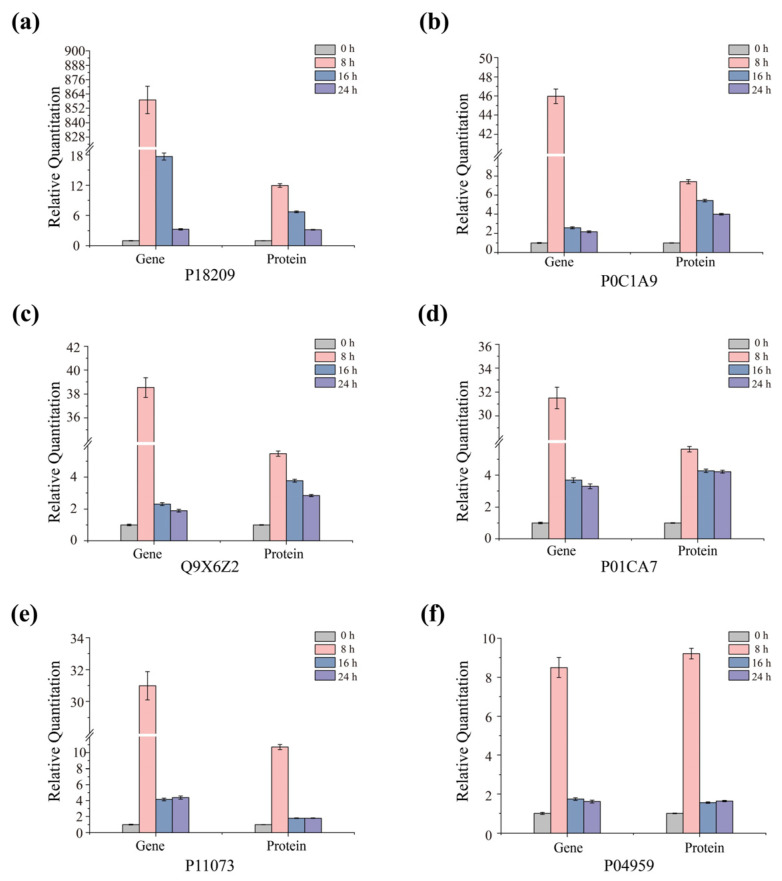
qRT-PCR analysis of key selected transcripts. The differential expression patterns of the six selected enzymes (P18209, P0C1A9, Q9X6Z2, P01CA7, P11073, and P04959) with significant differences in protein expression during the degumming process were verified by quantitative real-time PCR. The mRNA levels at different growth periods were presented relative to the 16S rRNA internal control, with the ratio at 0 h arbitrarily set as 1. Corresponding protein abundance profiles at different growth periods are provided for comparison. The error bars represent the standard deviation derived from six independent mRNA quantitative replicates. (**a**) P18209; (**b**) P0C1A9; (**c**) Q9X6Z2; (**d**) P01CA7; (**e**) P11073; (**f**) P04959.

To investigate whether the observed changes in the proteome correlate with corresponding changes at the transcriptome level, we screened differentially expressed proteases through functional annotation of differentially expressed genes. Subsequently, based on *p*-value < 0.05 and fold change (FC) > 2, we selected the top six genes with significant differential expression. Using quantitative real-time polymerase chain reaction (qRT-PCR), we measured the expression levels of six genes associated with key steps in the de-gumming process. These genes exhibited significant differences in protein expression levels. The results indicated that the mRNA expression patterns of these genes were consistent with the trends obtained from proteome analysis. This consistency suggests a positive correlation between transcriptional regulation and translational regulation of the selected targets. Specifically, the upregulation or downregulation trends of the genes were highly consistent with the changes in protein abundance (Figure 5). Relative quantitative analysis of the transcriptional levels (Gene) and corresponding protein expression levels (Protein) of the six key genes in the *D. dadantii* strain DCE-01 at different degumming time points (0 h, 8 h, and 24 h). The results showed that the mRNA expression patterns of these genes were consistent with the trends obtained from proteome analysis. This consistency indicates a positive correlation between the transcriptional regulation and translational regulation of the selected targets [33]. Specifically, the upregulation or downregulation trends of the genes were highly consistent with the changes in protein abundance (Figure 5). Genes such as P18209, P0C1A9, Q9X6Z2, P11073, and P04959, along with their corresponding proteins, showed significantly high expression levels at the early stage of degumming (8 h), indicating that the strain rapidly synthesizes a large amount of related enzymes at the beginning of degumming to rapidly decompose the inter-fiber gum; P0C1A7 and its corresponding protein also showed high expression levels at 8 h, but decreased at 24 h. As the degumming time extended to 24 h, the expression levels of these genes and proteins generally showed a downward trend, but remained at a relatively high level. For example, the gene and protein expression levels of P18209 reached a peak at 8 h and then declined at 24 h. The trend of gene and protein expression levels of P04959 was similar to that of P18209, reaching a peak at 8 h. The results of the entire graph were in good agreement with the proteomics analysis results. The upregulation or downregulation trends of the transcriptional levels (mRNA) were highly consistent with the trends of protein abundance changes.

These findings support the view that the observed changes in the protein profile under experimental conditions are largely driven by dynamic changes at the transcriptional level. This demonstrates that the *D. dadantii* strain DCE-01 precisely regulates gene expression, thereby controlling protein synthesis, to adapt to different stages of the hemp degumming process and ensure the efficient synergy of degumming enzymes. These findings support the view that the observed changes in the protein profile under experimental conditions are largely driven by dynamic changes at the transcriptional level. This proves that the *D. dadantii* strain DCE-01 precisely regulates gene expression, thereby controlling protein synthesis, to adapt to different stages of the hemp degumming process and ensure the efficient synergy of degumming enzymes.

## 4. Conclusions

This study performed a multi-dimensional analysis to elucidate the biological degumming mechanism of ramie fibers by *D. dadantii* strain DCE-01. At the macroscopic level, SEM observations revealed a gradual transformation from densely adhered fiber bundles covered with non-cellulosic residues at 0 h to completely separated and clean individual fibers at 24 h. This morphological evolution was consistent with the quantitative chemical analysis, which showed a steady increase in fiber weight loss from approximately 15% to 30% and a corresponding decrease in residual gum content from about 26% to 18%. The process, while efficient, was accompanied by a reduction in fiber tensile strength, indicating that degumming duration should be optimized to balance gum removal and fiber integrity. At the microscopic level, quantitative proteomics demonstrated that degumming by strain DCE-01 involves a dynamic, stage-specific, and synergistic enzymatic network. Early induction of pectate lyases (e.g., PelD, P18209) facilitated rapid pectin degradation, followed by sustained expression of hemicellulases (e.g., β-galactosidase, C8WV58) in later stages to remove residual hemicellulose. This coordinated enzyme regulation underscores a precise, multi-enzyme catalytic system underlying efficient biological degumming.

Compared with previously reported results from related fiber-degrading microorganisms, similar to other *Dickeya* strains, the strain DCE-01 proteome showed strong enrichment in pectinases, hemicellulases, and proteases, which are known to be the key enzymes in pectin and hemicellulose degradation during degumming [34]. However, the temporal dynamics observed here revealed a more coordinated activation of carbohydrate metabolism, amino-acid biosynthesis, and redox regulation pathways, suggesting a tighter coupling between energy generation and enzyme secretion than previously described [35]. In contrast to *Bacillus* species that primarily rely on extracellular pectinases for degumming [36], *D. dadantii* strain DCE-01 exhibited broader metabolic plasticity, involving both oxidative stress management and branched-chain amino-acid biosynthesis, consistent with its high efficiency in ramie fiber processing [37]. These findings collectively indicate that strain DCE-01 possesses a unique enzymatic and metabolic adaptation pattern that distinguishes it from other degumming bacteria, providing a valuable theoretical foundation for future strain engineering and process optimization [38].

## Data Availability

The original contributions presented in this study are included in the article/Supplementary Material. Further inquiries can be directed to the corresponding authors.

## Supplementary Materials

The following supporting information can be downloaded at https://www.mdpi.com/article/10.3390/polym17243284/s1. Table S1: Fiber diameters at different degumming time points; Table S2: Oligonucleotide primers for qRT-PCR.
