# Supplementary material for: The Temporal Extracellular Proteomics Analysis Reveals the Expression Patterns of Functional Enzymes Involved in Ramie Degumming by Dickeya dadantii Strain DCE-01
Source: Polymers (Basel). 2025 Dec 11;17(24):3284. doi: 10.3390/polym17243284 (PMC12736550; doi:10.3390/polym17243284)
Supplement: Supplementary file 1 [file polymers-17-03284-s001.zip › polymers-3929497-supplementary.pdf]

The temporal extracellular proteomics analysis reveals the expression patterns of functional enzymes involved in ramie degumming by *Dickeya dadantii* strain DCE-01

Yuqin Hu [1], Mingqiang Gao [2], Xiang Zhou [1], Lifeng Cheng [1], Guoguo Xi [1], Si Tan [1], Wei Zhou [1], Zishu Chen [1], Zhenghong Peng [1], An Wang [3], Shengwen Duan [1] *, Qi Yang [1] *

[1] Institute of Bast Fiber Crops, Chinese Academy of Agriculture Sciences, Changsha 410205, China;

[2] Songtao National Middle School of Guizhou Province，Song tao 554100, China；

[3] State Key Laboratory of Rice Biology and Breeding, China National Rice Research Institute, Hangzhou 310006, China；

82101232017@caas.cn (Y.H.); gmq18785604146@163.com (M.G.); 821012430018@caas.cn (X.Z.); chenglifeng@caas.cn (L.C.); xiguoguo@caas.cn (G.X.); 15116005853@163.com (S.T.); zhouwei@caas.cn (W.Z.); chenzishu@caas.cn (Z.C.); pengzhenghong@caas.cn (Z.P.); anwang010828@163.com (A.W.)

* Correspondence: duanshengwen@caas.cn (S.D.); yangqi@caas.cn (Q.Y.)

Table S1. Fiber diameters at different degumming time points (μm)

Degumming time	0h	8h	16h	24h
1	34.738	32.535	25.411	24.23
2	33.821	31.925	30.605	28.554
3	31.932	29.168	32.828	22.574
4	29.223	26.133	27.683	25.873
5	32.746	27.847	26.776	22.478
Average	32.492	29.5216	28.6606	24.7418

Table S2. Oligonucleotide primers for qRT-PCR

Primers	Genotype/description	Source/reference
16S-F	ACTCCTACGGGAGGCAGCAG	This work
16S-R	ATTACCGCGGCTGCTGG	This work
P11073-F	ATTAAGAAATCCTCCGACGTGG	This work
P11073-R	CACGGTGACATAGTCCGAAGC	This work
P04959-F	CAGCTCTGGTCTGAACGTGC	This work
P04959-R	CTTTGGTATCCGCCGTCC	This work
P0C1A9-F	CCTGAACACCAGTATGGATAACC	This work
P0C1A9-R	TGTGCGTCGGTCAGTTGC	This work
P0C1A7-F	CCGCAGAAAGTGGTGATTGA	This work
P0C1A7-R	TGCCAAATGCGACAGAGC	This work
Q9X6Z2-F	CGAAAGCCAGTTCCGAGTGTA	This work
Q9X6Z2-R	GCCGCTGTCGCAGTGAAT	This work
P18209-F	ACAGCAATGGCTCACAGGACT	This work
P18209-R	GATACGGGTAGACGCTGTGCT	This work
